# Canopy disturbance and gap partitioning promote the persistence of a pioneer tree population in a near‐climax temperate forest of the Qinling Mountains, China

**DOI:** 10.1002/ece3.5319

**Published:** 2019-06-06

**Authors:** Yaoxin Guo, Peng Zhao, Ming Yue

**Affiliations:** ^1^ Key Laboratory of Resource Biology and Biotechnology in Western China (Ministry of Education) Northwest University Xi'an China

**Keywords:** canopy gap, regeneration, shade tolerance, tree life history, tree‐species coexistence

## Abstract

An unresolved question of temperate forests is how pioneer tree species persist in mature forests. In order to understand the responsible mechanisms, we investigated a near‐climax mixed temperate forest dominated by *Betula albosinensis* in the Qinling Mountains of China. Through establishing four 50 m × 50 m plots, we examined the canopy disturbance characteristics and its effects on tree recruitments. We further test the intra‐ and interspecific effects on the recruitment of *B. albosinensis*. The obtained data demonstrated canopy disturbance was frequent but most small‐sized. The canopy gaps are caused mainly by adult *B. albosinensis* by snapping. The regeneration of coexistent tree species shows a distinct preference for gap size. *B. albosinensis* were clumped at the juvenile stage and small scales. *B. albosinensis* juveniles were positively associated with *B. utilis* juveniles and negatively associated with the conspecific and *B. utilis* large trees. In addition, *B. albosinensis* juveniles showed negative associations with contemporary other tree species. Our results suggested that canopy disturbance caused by canopy trees and gap partitioning among the coexistent tree species are important for the persistence of the mixed forest. As a main gapmaker, *B. albosinensis* appear to develop a self‐perpetuating life‐history trait and allow them to persist.

## 1 INTRODUCTION

Shade tolerance is a key factor in determining the persistence of tree population during the successional dynamics of most forests. Generally, light‐demanding pioneer trees are prone to be replaced by shade‐tolerant trees along the succession (Huston & Smith, 1987; Walters & Reich, 1996). However, some mature and old‐growth temperate forests in the Northern Hemisphere present a mixed canopy of tree species with different shade tolerances (Henry & Aarssen, 2001; Papaik & Canham, 2006; Taylor et al., 2006). An unresolved question concerning the dynamics of these forests is the unexpected persistence of shade‐intolerant tree species in the forest canopy. Species‐specific differences in patterns of tree regeneration, longevity, and seed dispersal have all been recognized to be relevant to the coexistence of tree species with different shade tolerances because interspecific differences in life‐history traits induce the shifts in species composition and dominance in response to local disturbance regimes (Gutiérrez et al., 2008; Taylor & Qin, 1988). However, the correspondence between disturbance regimes and species‐specific life‐history traits is not well understood.

In addition, inter‐ and intraspecific interactions are considered fundamental ecological processes regulating population dynamics, and coexistence of tree species (Szwagrzyk & Szewczyk, 2001; Tilman, 1994). The establishment and survival of seedlings are greatly affected by neighboring conspecific and heterospecific adults (Barner, Hacker, Menge, & Nielson, 2016; Blaser, Sitters, Hart, & Edwards, 2013; Halpern & Lutz, 2013). Therefore, understanding the intra‐ and interspecific relationships among the different growth stages is critical for understanding the dynamics of tree population (Coates, Canham, Beaudet, Sachs, & Messier, 2003; Pacala & Deutschman, 1995; Van de Peer, Verheyen, Kint, Cleemput, & Muys, 2017). Negative density‐dependent competition caused by conspecific adults is found important in allowing the coexistence of tree species in mature forests (Bai et al., 2012; Das, Battles, Stephenson, & Mantgem, 2011; Kuninaga, Hirayama, & Sakimoto, 2015; Liang et al., 2016; Lutz et al., 2014). Such strong intraspecific negative feedback existing between canopy and understory trees can offset the effect of species asymmetry in competition ability and promote coexistence of multiple tree species with different life‐history traits (Du, Zhou, & Etienne, 2011; Woods, 1984).

Hardwood‐conifer mixed forests are widespread in warm temperate subalpine forests of China. Particularly in the subalpine range of the Qinling Mountains, the near‐climax hardwood‐conifer mixed forests, extending over a wide elevation range over 700 m, constitute one of the most important forest ecosystems of central China (Dang, Zhang, Zhang, Jiang, & Zhang, 2010; Qin et al., 2011; Wang, Franklin, Ren, & Ouellette, 2006). Intriguingly, most of these mixed forests are dominated by a pioneer tree species, *Betula albosinensis* Burk. The analysis of soil sporopollenin indicates that *B. albosinensis* have existed in these forests at least from the Late Quaternary (Zhu, 1991). Therefore, many historical records and investigation reports in the Qinling Mountains listed the forest as a climax forest (Yue, Dang, & Gu, 2000; Zhu, 1991). To date, however, the ecological mechanism responsible for the long‐term persistence of *B. albosinensis* in these mature forests has not been studied.

In the present work, therefore, we seek to understand the mechanism explaining the long‐term persistence of pioneer *B. albosinensis* in the canopy of mature mixed forest. First, we documented the characteristics of canopy disturbance and the regeneration patterns of canopy tree species in the mixed forest. Secondly, we examined how the conspecific and heterospecific adults affect the recruitment of *B. albosinensis*. Finally, we discuss the possible mechanism responsible for the persistence of *B. albosinensis* based on the given disturbance regime.

## 2 MATERIALS AND METHODS

### 2.1 Study site

**Figure 1:**
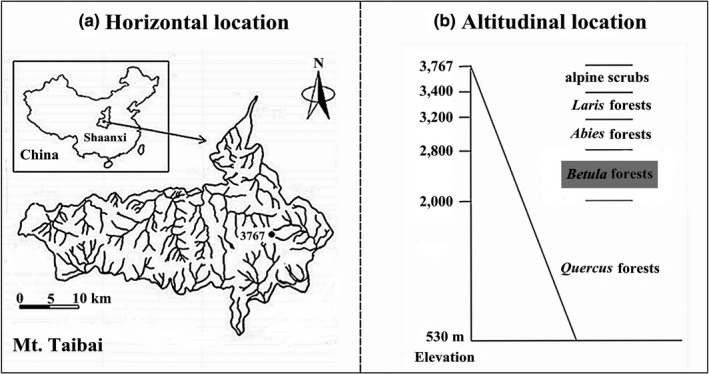
Location of study area in Shaanxi Province, China

This study was conducted in Mountain Taibai (33°49–34°10′N; 107°19′–107°58′E), the highest mountain of the Qinling Mountains, in Shaanxi, China (Figure 1a). Mt. Taibai spans from 530 to 3,767 m. The natural vegetation types are* Quercus* forests (<2,000 m), *Betula* forests (1,900–2,800 m), *Abies* forests (2,800–3200 m), *Larix* forests (3,000–3,400 m), and alpine scrubs (>3,400 m) along the altitudinal gradient (Figure 1b). This research focuses on the mature mixed forests dominated by *B. albosinensis* between 2,300 and 2,700 m, where the forest exhibits stable structure and tree composition. Besides *B. albosinensis*, the co‐occurring canopy tree species in these forests include *Quercus liaotungensis* Koidz.,* Pinus armandii* Franch., *Betula utilis* Don., and *Abies fargesii* Franch. along the altitude. These tree species span a wide range of reported shade tolerances. *B. albosinensis* and *B. utilis* are considered shade intolerant as pioneer species (Taylor et al., 2006; Taylor & Qin, 1988). *Q. liaotungensi and P. armandii* are considered less shade tolerant (Hou, Mi, Liu, & Ma, 2004). *A. fargesii* is considered shade tolerant (Dang et al., 2010; Taylor & Qin, 1988).

*B. albosinensis* forests have a cool‐warm moist climate, where the mean annual temperature is 4°C–10°C, and the mean annual rainfall is 750 mm–1,000 mm (Fu & Guo, 1994; Ren, Lin, & Yue, 2008). Historically, the *B. albosinensis* forests in the Qinling Mountains were selectively cut several times during the period 1790–1870 (Dang et al., 2010; Zhang, 1989). In the past century, however, human activities are rare due to the relatively difficult accessibility (Liu, Tang, Dai, Tang, & Cui, 2002). Therefore, the forest exhibits major natural disturbances (e.g., winds, floods, snowstorms; Dang et al., 2010) without anthropogenic disturbances. In mature *B. albosinensis* forest, most canopy *B. albosinensis* exceed 0.5 m in DBH (diameter at breast height). The largest tree recorded even exceeds 1 m in DBH and is nearly 100 years of age (calculated by equations between DBH and age).

### 2.2 Field methods

The spatial locations of trees provide signatory information for inferring inter‐ and intraspecific relationships in field conditions (Gray & He, 2009); therefore, analyzing the spatial patterns of trees is an effective method of investigating inter‐ and intraspecific interactions of tree species in the absence of growth data (Browning, Franklin, Archer, Gillan, & Guertin, 2014; Guo et al., 2015). In the present study, we sampled four stands to represent the typical floristic composition of *B. albosinensis* forest along the altitude (Table 1). Each stand was sampled with a 50 m × 50 m plot. Each plot was established on the consistent slop and microtopography to avoid the confounding effects of environmental heterogeneity on tree survival. Within each plot, only canopy tree species with more than 30 individuals were investigated to analyze spatial pattern. For each investigated tree species, all living stems greater than 5 cm in DBH were measured and mapped. Each tree species were divided into three growth stages according to DBH: juvenile (J) (5 cm ≤ DBH < 10 cm), medium (M) (10 cm ≤ DBH ≤ 25 cm), and large (L) (DBH > 25 cm).

**Table 1:** Characteristics of the four Plots in the mixed forest of Qinling Mountains, China

Characteristics	Plot I	Plot II	Plot III	Plot IV
Altitude (m)	2,397	2,418	2,526	2,663
Slop (°)	30	28	16	18
Aspect	NW	N	NW	N
Tree layer coverage (%)	60	65	75	70
Shrub layer coverage (%)	80	70	60	55
Herb layer coverage (%)	65	65	80	75
Basal area (m^2^/ha)	18.8	17.6	21.3	22.8
Main trees (ind./ha)	*B. albosinensis* (284) *P. armandii* (171) *Q. liaotungensis* (148)	*B. albosinensis* (366) *P. armandii* (283)	*B. albosinensis* (281) *P. armandii* (203) *B. utilis* (182)	*B. albosinensis* (273) *B. utilis* (192) *A. fargesii* (164)
No. of gaps	17	18	15	15
Sum areas of gaps (m^2^)	990	1,005	675	616
Averaged area of gaps (m^2^)	58.2	55.8	44.9	41.1
Maximum gap size (m^2^)	112	118	92	88
Gap area (as % of total area, %)	39.6	40.2	26.7	24.4
No. of gapmakers	28	27	38	38

In addition, canopy gaps within four plots were investigated. We considered gaps as all canopy openings with an area exceeding 20 m^2^ that appeared to have been formed by the death of one or more trees. The actual gap size was calculated with elliptical method based on the length (longest distance within the gap) and width (perpendicular to the length) for each gap (Runkle, 1981, 1982). At each canopy gap, the gap‐forming species (gapmakers) were identified and measured. At the same time, the gapmakers were categorized as: standing dead, snapped, uprooted, and branch fallen following Nakashizuka (1989). In addition, we recorded the regeneration of trees inside the expanded gap by direct counting seedlings (DBH < 5 cm). The expanded gap was defined as the actual gap plus the adjacent area extending to the bases of canopy trees surrounding the canopy gap; therefore, its benefit is that it includes area directly and indirectly influenced by the canopy opening (Runkle, 1982).

### 2.3 Data analyses

The *O*‐ring statistic was used to analyze the spatial patterns and associations of trees in each plot. Based on Ripley's K (Ripley, 1981) and mark correlation functions (Stoyan & Stoyan, 1994), the *O*‐ring statistics replaces the circles used for Ripley's K with rings and uses the mean number of neighbors in a ring of radius r and ring width around an individual, thus isolating specific distance classes (Wiegand & Moloney, 2004). The *O*‐ring statistics characterizes patterns by the frequency of points co‐occurring at a given distance, so it can analyze the spatial patterns derived from ecological processes easily and intuitively (Schurr, Bossdorf, Milton, & Schumacher, 2004; Wiegand & Moloney, 2004). The *O*‐ring statistics includes both univariate and bivariate statistics (Getzin et al., 2006). The univariate statistics are used to analyze the spatial pattern of one object, while the bivariate statistics are used to analyze the spatial association between two objects (pattern 1 and pattern 2). Following the notation by Wiegand and Moloney (2004), the bivariate *O*‐ring statistic *O*_12_(*r*) is calculated as:

(1) $${\hat{O}}_{12}^{w}\left(r\right)=\frac{\left(1/n_{1}\right)\sum_{i=1}^{n_{i}}{\text{Points}}_{2}\left[R_{1,}{}_{i}^{w}\left(r\right)\right]}{\left(1/n_{1}\right)\sum_{i=1}^{n_{i}}\text{Area}\left[R_{1,}{}_{i}^{w}\left(r\right)\right]}$$

where *n*_1_ is the number of points of pattern 1; *R*_1_, ${}_{i}^{w}\left(r\right)$ the ring with radius *r* and ring width *w* centered in the *i*th point of pattern 1; Points_2_[*X*] (Equation 2) counts the points of pattern 2 in a region X; and the operator Area[*X*] (Equation 4) determines the area of the region *X*.

(2) $${\text{Points}}_{2}\left[R_{1,}{}_{i}^{w}\left(r\right)\right]=\sum_{\mathrm{all}x}\sum_{\mathrm{all}y}S\left(x,y\right)P_{2}\left(x,y\right)I_{r}\left(x_{i},y_{i},x,y\right)$$

where (*x_i_*, *y_i_*) are the coordinates of the *i*th point of pattern 1; *S*(*x*, *y*) is an identifier to each cell (*x*, y) (*S*(*x*, *y*) = 1 if a cell with coordinates (*x*, *y*) is inside the boundaries of the study region, otherwise *S*(*x*, *y*) = 0); *P*_2_(*x*, *y*) gives the number of points of pattern 2 lying within the cell; and the counter variable *Ir* (Equation 3) defines the circle with radius r that is centered at the *i*th point of pattern 1:

(3) $$I_{r}^{w}\left(x_{i},y_{i},x,y\right)=\left\{\begin{array}{cc} 1 & \text{if}\;r-\frac{w}{2}\leq \sqrt{{\left(x-x_{i}\right)}^{2}+{\left(y-y_{i}\right)}^{2}}\leq r+\frac{w}{2}\\ 0 & \text{otherwise} \end{array}\right.$$

(4) $$\text{Area}\left[R_{1,}{}_{i}^{w}\left(r\right)\right]=z^{2}\sum_{\text{all}\;x}\sum_{\text{all}\;y}S\left(x,y\right)I_{r}\left(x_{i},y_{i},x,y\right)$$

where *z* ^2^ is the area of one cell. The univariate *O*‐ring statistic *O*(*r*) is calculated by setting pattern 2 equal to pattern 1.

In our study, univariate *O*‐ring statistic was used to analyze the spatial patterns of *B. albosinensis* at the different growth stage, respectively, and bivariate *O*‐ring statistic was used to examine the intraspecific and interspecific associations in spatial distribution. Different null models were selected for various analyses because the use of an inappropriate null model may lead to a misinterpretation of spatial patterns (Goreaud & Pélissier, 2003). For the univariate *O‐*ring statistic, complete spatial randomness was selected as the null model because there was no broad‐scale heterogeneity in the stand caused by exogenous factors. For the bivariate *O‐*ring statistic, data were analyzed using an antecedent‐condition null model that keep the older stages fixed and randomize the earlier ones because the different growth stages of trees were not achieved at the same time but in sequence.

In the univariate statistic, the values of *O*(*r*) within the confidence intervals indicate a random distribution at a given distance, whereas the values of *O*(*r*) above the upper (or below the lower) limit of the confidence envelop indicate a clumped (or a regular) distribution. In the bivariate statistic, values of *O_12_*(*r*) within the confidence intervals indicate an absence of interaction at a given distance, whereas the values of *O_12_*(*r*) above the upper (or below the lower) confidence intervals indicate a positive (or a negative) association. The 99% confidence envelopes were calculated from the highest and lowest values obtained from 99 simulations of the null model. The *O‐*ring statistics were computed using Programita software (Wiegand & Moloney, 2004).

## 3 RESULTS

### 3.1 Population structure

A total of 5 canopy tree species were investigated within the sampled mixed forests (Table 1). *B. albosinensis* dominated all the plots with the highest density, accounting for 47%, 56%, 42%, and 43%, respectively. In addition, *P. armandii* accounted for approximate 28% in Plot I, 43% in Plot II, and 31% in Plot III and *B. utilis* accounting for 27% in Plot III and 31% in Plot IV. *Q. liaotungensis* (25%) and *A. Fargesii* (26%) were only found in Plot I and Plot IV, respectively**.**

**Figure 2:**
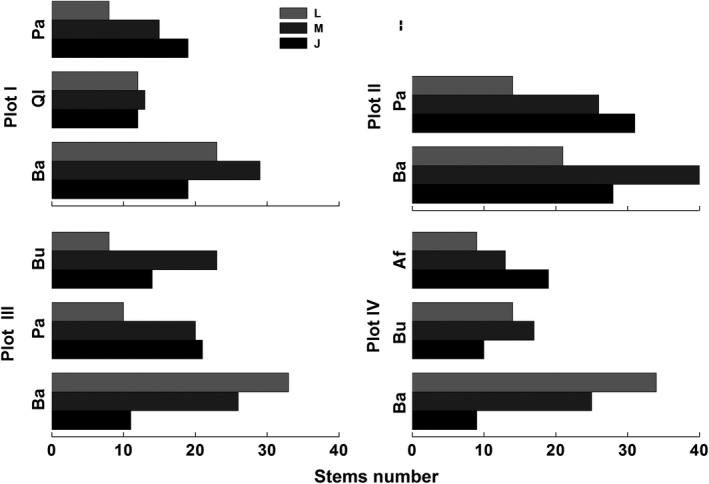
Size distributions of canopy tree species in the four Plots of the mixed forest. “J,” “M,” and “L” represent juvenile, medium‐sized, and large‐sized trees, respectively; Ba, *Betula albosinensis*; Ql, *Quercus liaotungensis*; Pa, *Pinus armandii*; Bu, *Betula utilis*; Af, *Abies fargesii*

*B. albosinensis* showed different size structures among the Plots (Figure 2). In Plots I and II, *B. albosinensis* was dominated by the medium‐sized individuals, while large‐sized trees were dominant and young trees were scarce in Plots III and IV. *P. armandii* and *A. fargesii* were prevalent at the young stage, and relatively few were large‐sized. *B. utilis* was dominated by the medium‐sized trees, while *Q. liaotungensis* showed an even distribution among the different growth stages.

### 3.2 Gap formation and regeneration

**Figure 3:**
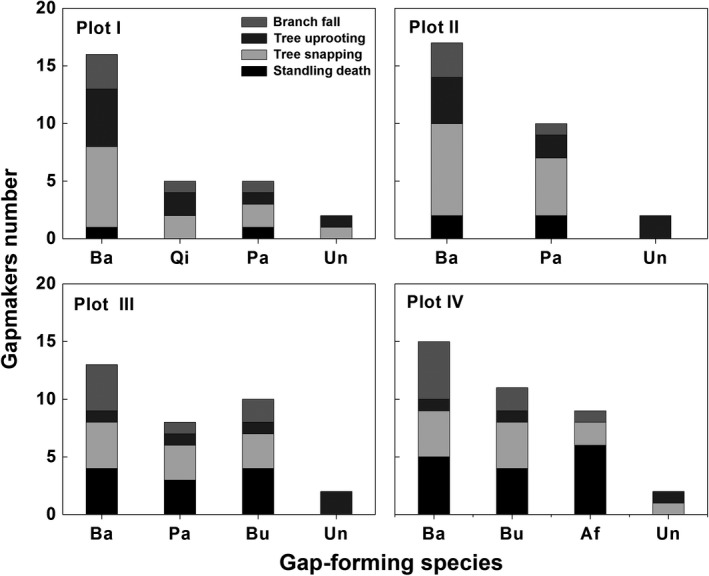
Mechanisms of gap formation in the four plots of the mixed forest. Ba, *Betula albosinensis*; Ql, *Quercus liaotungensis*; Pa, *Pinus armandii*; Bu, *Betula utilis*; Af, *Abies fargesii*; Un, unidentified

**Figure 4:**
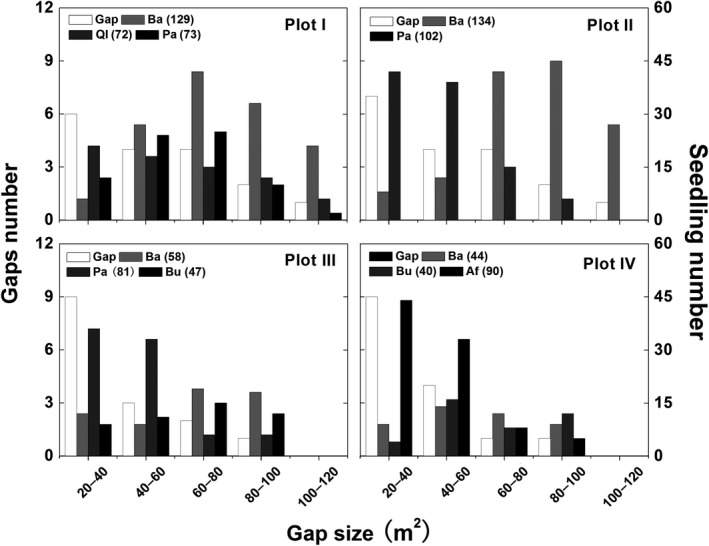
Gap size distribution and regeneration in the four plots of the mixed forest. Ba, *Betula albosinensis*; Ql, *Quercus liaotungensis*; Pa, *Pinus armandii*; Bu, *Betula utilis*; Af, *Abies fargesii*

A total of 65 gaps were measured, covering 32.7% of the total investigated area. Plot II showed a highest gap density and area, followed by Plot I, Plot III, and Plot IV (Table 1). Most gaps are small‐sized in the forest, and only, two gaps exceeded 100 m^2^, which were found in Plots I and II (Figure 4). Within the 65 gaps, a total of 131 gapmakers were identified (Table 1). The mechanism of gap formation was mainly tree snapping which accounted for 36% of total gap formation, followed by standing death (25.2%), tree uprooting (19.6%), and branch fall (18.9%). In Plots I and II, tree snapping and uprooting were important in gap formation, while tree snapping and uprooting and standing death were in gap formation of in Plots III and IV (Figure 3). *B. albosinensis* was the most common gapmaker (48%) in four Plots (Figure 3). *P. armandii* (18%) was common in Plots I, II, and III. *B. utilis* (16%) was common in Plots III and IV. *A. fargesii* (7.8%) were also important gapmakers in the Plot IV. *Q. liaotungensis* (4.0%) were infrequent gapmakers in their Plot I. In addition, 6.3% of gapmakers were highly decomposed and unidentified.

**Figure 5:**
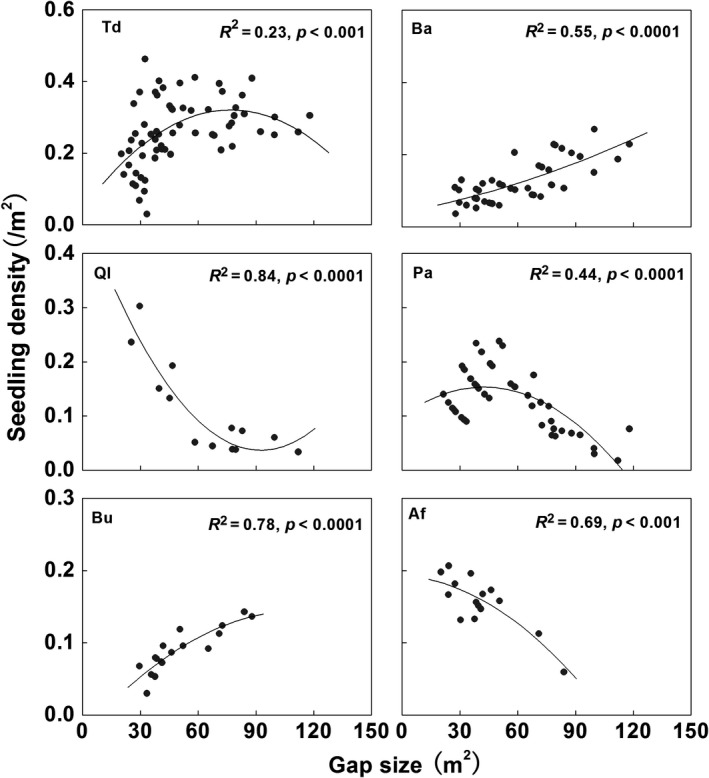
Relationships of seedling density among the different tree species with canopy gap sizes. Ba, *Betula albosinensis*; Ql, *Quercus liaotungensis*; Pa, *Pinus armandii*; Bu, *Betula utilis*; Af, *Abies fargesii;* Td, Total density

A total of 870 tree seedlings were found in the gaps. *B. albosinensis* seedlings were most abundant among the tree species, which accounted for 47.1% in Plot I, 56.8% in Plot II, 31.2% in Plot III, and 25.3% in Plot IV. In addition, *P. armandii* seedlings were also abundant in Plots II and III. *A. fargesii* seedlings were abundant in Plot IV. The total and species‐specific seedling density showed significant relationships with gap size (Figure 5). Seedling density of total and *P. armandii* both showed a parabola trend with the increased gap size. Seedling density of *B. albosinensis* and *B. utilis* increased significantly with gap size. *Q. liaotungensis* and *A. fargesii* decreased significantly with gap size.

### 3.3 Spatial patterns and relationships

**Figure 6:**
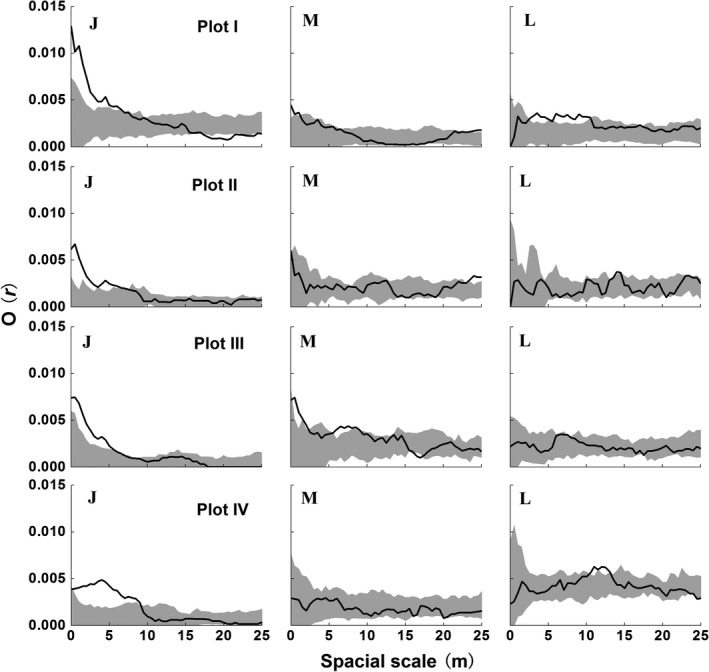
Spatial distribution pattern of *B. albosinensis* population at different growth stages in the four Plots of the mixed forest. The solid line indicates ring statistic, *O*(*r*); the gray‐filled area indicates the upper and lower limits of the 99% confidence envelope of the null model. “J,” “M,” and “L” represent juvenile, medium‐sized, and large‐sized trees, respectively

**Figure 7:**
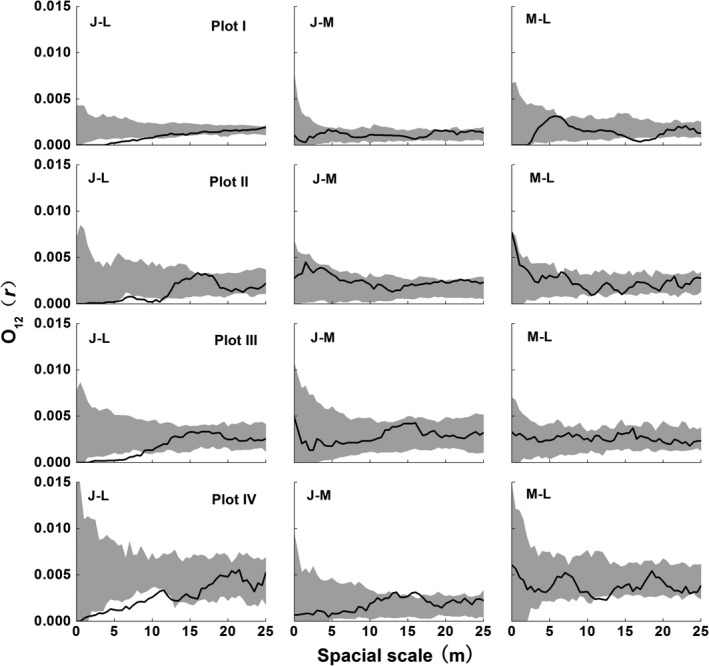
Spatial association among the different growth stages of *B. albosinensis* population in the four Plots of the mixed forest. The solid line indicates ring statistic, *O*(*r*); the gray‐filled area indicates the upper and lower limits of the 99% confidence envelope of the null model. “J,” “M,” and “L” represent juvenile, medium‐sized, and large‐sized trees, respectively

The spatial pattern of *B. albosinensis* varied along the life cycle (Figure 6). Although *B. albosinensis* juveniles had different density among the four Plots, spatial patterns were similarly clumped at nearly 0–10 m scales. Medium‐sized *B. albosinensis* were only clumped at scales of <10 m in Plot III. Large‐sized *B. albosinensis* showed stochastic distribution at most scales except for clustered distribution at scales of 4–10 m in Plot I. In the four Plots, young *B. albosinensis* showed negative associations with large conspecific adults at scales of 0–10 m, whereas medium‐sized *B. albosinensis* were spatially independent with two other size classes (Figure 7).

In addition, some notable interactions were found between *B. albosinensis* juveniles with the nonconspecific trees (Table 2). Juvenile *B. albosinensis* were positively associated with *B. utilis* juveniles and negatively with large *B. utilis* adults at small scales. In addition, juvenile *B. albosinensis* were negatively associated with *Q. liaotungensis, P. armandii,* and *A. fargesii* juveniles at small scales and unrelated to their adults.

**Table 2:** Spatial associations of *B. albosinensis* juveniles with coexistent tree species in the four Plots of the mixed forest

Plots	Species associations	Scale (m)				
		0–5	5–10	10–15	15–20	20–25
Plot I	Ba_J‐Ql_J	−	−	r	r	r
	Ba_J‐Pa_J	−	−	r	r	r
	Ba_J‐Ql_M	r	r	r	r	r
	Ba_J‐Pa_M	r	r	r	r	r
	Ba_J‐Ql_L	−	−	r	r	r
	Ba_J‐Pa_L	r	r	r	r	r
Plot II	Ba_J‐Pa_J	−	−	−	r	r
	Ba_J‐Pa_M	r	+	+	r	r
	Ba_J‐Pa_L	r	r	r	r	r
Plot III	Ba_J‐Pa_J	−	−	r	r	r
	Ba_J‐Bu_J	+	+	+	r	r
	Ba_J‐Pa_M	r	+	r	r	r
	Ba_J‐Bu_M	−	r	r	r	r
	Ba_J‐Pa_L	r	r	r	r	r
	Ba_J‐Bu_L	−	−	r	r	r
Plot IV	Ba_J‐Bu_J	+	+	r	r	r
	Ba_J‐Af_J	−	−	−	r	r
	Ba_J‐Bu_M	r	r	r	r	r
	Ba_J‐Af_M	r	r	+	r	r
	Ba_J‐Bu_L	−	−	r	r	r
	Ba_J‐Af_L	r	r	r	r	r

Note

“+” indicates positive association, “**−**” indicates negative association, and “r” indicates random relationship; “J,” “M,” and “L” represent juvenile, medium‐sized, and large‐sized trees, respectively.

Abbreviations: Ba, *Betula albosinensis*; Ql, *Quercus liaotungensis*; Pa, *Pinus armandii*; Bu, *Betula utilis*; Af, *Abies fargesii*.

## 4 DISCUSSION

### 4.1 Disturbance and regeneration dynamics

In our study, a total of 65 canopy gaps were identified within the total of 1.0 ha forest area, indicating that canopy disturbance is abundant in the present forests. However, most gaps were <100 m^2^, which was consistent with other reports in temperate forests that small gaps dominate natural disturbance regimes (Fukui, Hirao, Murakami, & Hirakawa, 2011; Sipe & Bazzaz, 1994). In the gap formation, tree snapping was the most common mechanism, which is consistent with the mechanism of canopy gap formation in mixed hardwood‐conifer forests in Foping National Natural Reserve of the Qinling Mountains where 40% of gaps are tree snap types (Wang et al., 2006). In the mixed *B. albosinensis* forests, seasonal wind and snow are the main natural disturbance types (Dang et al., 2010; Qin et al., 2011). Most canopy trees are susceptible to wind events and snow accumulation; therefore, tree snapping is most frequent after these disturbance events. Besides tree snapping, we found that uprooting was more common in Plots I and II, and standing death was more common in the Plots III and IV. The difference might be attributed to the change in slope between the Plots. In our study, Plots I and II were located in steep sites, whereas Plots III and IV had gentle slopes (see Table 1). Steep topography develops shallow soils and confronts intense seasonal wind and flood, which may increase the likelihood of tree uprooting. In contrast, the trees growing on gentle slopes may seldom uproot, but they are prone to become standing death due to disease in the poorly‐drained soils (Sefidi, Mohadjer, Mosandl, & Copenheaver, 2011). In addition, *Betula* trees are the most common gapmakers in the mixed forest; in particular, *B. albosinensis* accounted for 48% of the gapmakers. The high mortality rate of *Betula* trees also has been found in the other hardwood‐conifer forests of China (Taylor, Jinyan, & ShiQiang, 2004; Wang et al., 2006). As pioneer trees, *Betula* trees grow rapidly and develop fragile stems and large crown (Lusk, 1995). The way *Betula* grows makes them susceptible to local disturbances, such as seasonal wind and snow accumulation. Therefore, canopy gaps created by *Betula* trees were prevalent in the present forest.

In our study, seedling density was largest in the gaps of medium size, suggesting medium‐sized gap may be more important for the regeneration of most tree species. However, most tree species showed species‐specific regeneration pattern in relation to gap size (Figure 5). The recruits of *B. albosinensis* and *B. utilis* increased significantly with gap size, which is consistent to the previous findings that an environment with much light and exposed soil are important for the establishment and survival of *Betula* sp. (Nakamura, 1985; Taylor & Qin, 1988; Seiwa & Kikuzawa, 1996; Guo et al., 2013). *Betula* sp. has life‐history traits that are associated with large gap specialists, such as shade intolerance, long‐distance seed dispersal, and fast‐growing seedlings, which contribute to their regeneration in large‐sized gaps. In our study, therefore, Plots I and II have more abundant *B. albosinensis* seedlings than Plots III and IV since Plots I and II have more large‐sized gaps (Figure 4). In contrast, densities of *Q. liaotungensis* and *A. fargesii* seedlings decreased significantly with gap size. Previous research suggested that *Quercus* seedling response to light availability varies among species (Sevillano, Short, Grant, & O'Reilly, 2016; Truax, Lambert, & Gagnon, 2000). Nevertheless, most researchers have found positive effects of increased light on *Q. liaotungensis* seedling establishment. For example, Li and Ma (2003) found that seedling recruitment of *Q. liaotungensis* was significantly better in gaps than in shaded understory. However, *Q. liaotungensis* seed fall occurs under and near the crown of the parent tree, which does not allow them to reach large‐sized gaps, although the environment is favorable for establishment (Mi & Hou, 2009). Thus, *Q. liaotungensis* seedlings may be limited to the small‐sized gaps due to limited dispersal. It can explain the decline of *Q. liaotungensis* seedling along the increased gap size. Compared with *Q. liaotungensis, A. fargesii* is considered to be more shade tolerant and have lower light requirements for regeneration than hardwood trees although it also depends on gaps to keep the population sustainable and stable (Dang et al., 2010). Fu, Liu, and Xiong (2010) found the growth and distribution of *A. fargesii* seedlings decreased in the order of gap size in the Shennongjia National Nature Reserve of China. Similarly, *A. fargesii* had higher seedling density in the small‐sized gaps in our study, suggesting microenvironment under small‐sized gaps is more suitable for the recruitment of *A. fargesii*. Different than in other canopy trees, the seedling density of *P. armandii* peaked in the medium‐sized gap. Many published studies in China have found that *P. armandii* is less shade tolerant (Lan, Lei, & An, 2005; Yu et al., 2014). In addition, moist soil is also necessary for the establishment of *P. armandii* seedling (Gao, 1991). Soil moisture is variable with gap size. In general, intermediate‐sized gaps can maintain wetter soil than small‐ and large‐sized gaps because the total input of rainfall in small gap is less due to the interception loss and the large gap suffer increased evaporation and transpiration (Gray, Spies, & Easter, 2002; Ochiai, Okuda, & Sato, 1994). Therefore, the moist soil of the medium‐sized gap might be possible to influence on the regeneration of *P. armandii*.

### 4.2 Intra‐ and interspecific relationships

Our results showed that, with a few exceptions (e.g., clustered distribution of large‐sized trees at scales of 4–10 m in plot I), there was an evident shift in spatial distribution from aggregation to randomness along the life cycle for *B. albosinensis* (Figure 6). The nonrandom mortality driven by intraspecific competition is also observed in other temperate forests (Duncan, 1991; Szwagrzyk & Czerwczak, 1993), supporting that density‐dependent mortality is a prevailing mechanism in the dynamics of temperate tree populations. In addition, we also found a negatively spatial relationship of juvenile versus large *B. albosinensis* (Figure 7), suggesting the recruitment of *B. albosinensis* was affected by conspecific large trees. It is inconsistent with the findings of Hou et al. (2004) that young *Betula dahurica* show positive associations with live adults. The different intraspecific association between the two *Betula* trees may be attributed to the different regeneration strategies of them. *B. dahurica* generally regenerate through sprouting, whereas seedling recruitment was an important regeneration strategy for *B. albosinensis*. We infer that this local‐scale conspecific repulsion of *B. albosinensis* has resulted from intraspecific competition because Hou et al. (2004) found that there was a positive association of small size dead *Betula* trees with adult conspecifics at small scales in a *Quercus‐Betula* forest of northern China, suggesting that mortality of these small trees was due to intraspecific competition for resources with larger surrounding trees. Therefore, the negative canopy–understory interaction of *B. albosinensis* might be caused by intraspecific competition for light in consideration of the shade‐intolerant characteristic.

Spatial associations among species are indicative of interspecific interaction in the past (Rejmánek & Lepš, 1996). In the present study, juveniles of *B. albosinensis* were positively associated with young *B. utilis* and negatively associated with large *B. utilis* at small scales. Both as pioneer species, *B. albosinensis* and *B. utilis* have similar regeneration microsites, which may be responsible for their positive association in space. Just like the intraspecific canopy–understory competition of *B. albosinensis*, the similar life‐history feature also causes the recruitment of *B. albosinensis* repulsed by adult *B. utilis* and result in intraspecific canopy–understory competition, which can explain the negative association between young *B. albosinensis* and adult *B. utilis*. In addition, young *B. albosinensis* showed negative associations with the juveniles of the other three canopy trees. Besides *B. utilis,* the regeneration of tree species shows a distinct preference for gaps of different size (see Figure 5), suggesting that they have different microsites for regeneration. Hence, differential patterns of regeneration among these species may be responsible for their negative associations in regenerating site.

### 4.3 What responsible for the persistence of B. albosinensis

Recruitment limitation is a key factor affecting the persistence of *B. albosinensis* in the closed forests over the long term; it is therefore generally considered to be an unstable early‐successional species. However, the viewpoint is based on the inherent life‐history characteristics without considering of variable local environmental condition. The direction of community succession depends on the comprehensive influence of species life history and specific abiotic condition, and environmental condition contributes more at some time (Clements, 1936; Connell & Slatyer, 1977). In the mixed forests of present study, although the current recruitment of *B. albosinensis* was deficient (indicated by the current unimodal pattern in size distribution), the long residence time of *B. albosinensis* (as inferred from the large size of these trees) in the canopy and its frequency as a gapmaker seems to create conditions that favor its own regeneration. In addition, the spatial segregation of seedlings of different tree species along the gap size gradient suggests the partitioning of gaps by size may play an important role in maintaining the stability of the mixed canopy. Gap partitioning is considered to be an important mechanism in maintaining the coexistence of multiple tree species since it can decrease the interspecific competition (Nakashizuka, 2001). In mature forest, interspecific competition in the seedling stage is crucial to the survival and growth of the pioneer species seedlings. Negative associations in the seedling stage of *B. albosinensis* with coexistent species suggest gap partitioning can help them avoiding the interspecific competition, which may also contribute a lot to the long‐time persistence of *B. albosinensis*. Therefore, our results support the idea that long life spans, coupled with differences in seedling shade tolerance, and the creation of canopy gaps of different sizes by canopy trees are important factors for the nonequilibrium persistence of tree species mixtures (Gutiérrez et al., 2008; Lusk & Smith, 1998). Canopy disturbance and gap partitioning may redirect the fate of being replaced by *B. albosinensis* and allow them to persist in the mixed mature forest.

## CONFLICT OF INTEREST

No potential conflict of interest was reported by the authors.

## AUTHOR CONTRIBUTIONS

YG and MY conceived and designed the experiments; YG and PZ conducted field work and analyzed the data; YG wrote the manuscript.

## Data Availability

Sampling locations and gap investigational data: Dryad doi: https://doi.org/10.5061/dryad.4b562r8.
